# 单中心394例广泛期小细胞肺癌的一线化疗及生存分析

**DOI:** 10.3779/j.issn.1009-3419.2014.01.02

**Published:** 2014-01-20

**Authors:** 满姣 马, 孟昭 王, 燕 徐, 克 胡, 慧慧 刘, 龙芸 李, 巍 钟, 力 张, 静 赵, 华竹 王

**Affiliations:** 1 100730 北京，中国医学科学院北京协和医院呼吸内科 Department of Respiratory Medicine, Peking Union Medical College Hospital, Chinese Academy of Medical Sciences, Beijing 100730, China; 2 100730 北京，中国医学科学院北京协和医院肿瘤放疗科 Department of Oncological Radiotherapy, Peking Union Medical College Hospital, Chinese Academy of Medical Sciences, Beijing 100730, China

**Keywords:** 肺肿瘤, 广泛期, 生存, 影响因素, Lung neoplasms, Extensive-stage, Survival, Prognostic factors

## Abstract

**背景与目的:**

小细胞肺癌（small cell lung cancer, SCLC）是恶性程度极高的神经内分泌肿瘤，对放化疗敏感。目前，广泛期SCLC的一线标准化疗方案为铂类联合依托泊苷方案，但大多数接受一线化疗的患者在1年-2年内复发。一旦疾病复发，预后不良。本研究旨在研究广泛期SCLC总体和一线化疗的生存情况及其影响因素。

**方法:**

收集2001年2月-2011年12月经病理学或细胞学确诊为广泛期的SCLC患者394例，采用*Kaplan-Meier*法计算总生存时间（overall survival, OS）和无进展生存时间（progression-free survival, PFS）并绘制生存曲线，单因素及*Cox*回归多因素分析各种因素对生存期的影响。

**结果:**

全组中位OS为14.8个月，1年、2年、5年生存率分别为58.9%、27.2%、7.8%。全组OS与年龄（*P*=0.006）、ECOG评分（*P*=0.021）、肝转移（*P* < 0.001）、骨转移（*P* < 0.001）、是否化疗（*P* < 0.001）密切相关。一线化疗广泛期SCLC患者的中位OS为15.1个月，中位PFS为7.5个月。多因素分析结果显示一线化疗广泛期SCLC的OS与吸烟（*P*=0.041）、肝转移（*P* < 0.001）、骨转移（*P* < 0.001）、化疗疗程数（*P* < 0.001）相关；一线化疗PFS与吸烟（*P*=0.003）、肝转移（*P*=0.001）、骨转移（*P* < 0.001）、化疗疗程数（*P* < 0.001）相关。胸部放疗并非广泛期SCLC OS和PFS的独立影响因素。

**结论:**

年龄 < 60岁、体能状况好、无肝、骨转移的广泛期SCLC患者预后更好。广泛期SCLC患者应积极进行化疗，一线化疗的化疗疗效达到部分缓解-完全缓解有益于生存；适合的化疗疗程数目是4-6疗程。胸部放疗在广泛期SCLC治疗中的作用需要进一步研究。

## Abstract (en)

**Background and objective:**

Small cell lung cancer (SCLC) is the most malignant neuroendocrine tumor but highly sensitive to chemotherapy and radiotherapy. At present, the standard first-line chemotherapy regimen of extensive-stage SCLC is platinum combined etoposide regimen. However, most patients who receive first-line chemotherapy will relapse within one to two years. Once recurrent, it indicates poor prognosis. In this study, we analyzed the survival among all extensive-stage SCLC and patients who received first-line chemotherapy and determined prognostic factors.

**Methods:**

Total of 394 patients who were diagnosed as extensive-stage small cell lung cancer from February 2001 to December 2011 hospitalized in Peking Union Medical College Hospital were collected. *Kaplan-Meier* method was used to calculate the overall survival (OS) and progression-free survival (PFS). Univariate analysis and *Cox* regression analysis were used to detect the influence factors of survival.

**Results:**

The median OS of all extensive-stage small cell lung cancer was 14.8 months; 1-year, 2-year and 5-year survival rates were 58.9%, 27.2% and 7.8%, respectively. According to the results of univariate and *Cox* multivariate analysis, OS of extensive-stage SCLC was closely associated with age (*P*=0.006), ECOG PS (*P*=0.021), liver metastasis (*P* < 0.001), bone metastasis (*P* < 0.001) and chemotherapy (*P* < 0.001). The mortality risk of patients who didn't receive chemotherapy was 4.919 times higher than that who received; the mortality risk of patients without liver, bone metastasis was reduced by approximately 50 percent. The first-line chemotherapy was mainly EP (DDP+VP-16) or CE (CBP+VP-16) regimens (accounting for 82.8%) with 4-6 cycles. The median OS and PFS in first-line chemotherapy were 15.1 months and 7.5 months, respectively. The result of *Cox* regression analysis indicated that OS in first-line chemotherapy was remarkably related to smoking history (*P*=0.041), liver metastasis (*P* < 0.001), bone metastasis (*P* < 0.001), chemotherapy cycle number (*P* < 0.001); PFS was relevant with smoking history (*P*=0.003), liver metastasis (*P*=0.001), bone metastasis (*P* < 0.001), chemotherapy cycle number (*P* < 0.001). Thoracic radiotherapy was not an independent influence factor of OS and PFS in extensive-stage small cell lung cancer.

**Conclusion:**

The patients who were younger than 60-year old, with good KPS, absence of liver and bone metastasis had better prognosis. Patients should receive chemotherapy with first-line standard regimen (CE/EP regimen). It was beneficial to survival if the effect of first-line chemotherapy was SD or PR-CR and the proper chemotherapy cycle number was 4-6 cycles. The role of thoracic radiotherapy in extensive-stage small cell lung cancer needed to be investigated further.

小细胞肺癌（small cell lung cancer, SCLC）是恶性程度极高的神经内分泌肿瘤，属于肺癌的未分化型，约占所有肺癌的15%^[1]^。初诊时约60%-70%的SCLC患者属于广泛期，30%-40%属于局限期。广泛期SCLC中位生存时间约10个月-14个月，5年生存率为6.5%^[2, 3]^。近30年虽然有新的化疗药物出现，但广泛期SCLC患者生存情况的改善不明显。1981至2008年所有广泛期SCLC的Ⅲ期临床数据显示，生存时间每年只提高了0.63天^[4]^。本文回顾性地分析了在北京协和医院住院治疗并于2001年2月-2011年12月经病理学或细胞学确诊的394例广泛期SCLC患者的临床资料，研究广泛期SCLC的总体和一线化疗的生存情况及其影响因素，旨在为临床治疗广泛期SCLC和判断预后提供一定的参考依据。

## 1 资料与方法

### 1.1 研究对象

收集于2001年1月-2011年12月在北京协和医院住院治疗的SCLC患者的临床资料。所有患者需符合以下条件：①年龄 > 18周岁；②经细胞学或组织病理学检查确诊为SCLC；③经胸腹部增强CT、全身骨扫描、头颅增强MRI检查，根据美国退伍军人肺癌研究组（Veterans Administration Lung Study Group, VALG）制定的分期方法判定为广泛期SCLC；④病历资料完整。

### 1.2 研究方法

收集患者完整的临床资料，包括：患者的年龄、性别、吸烟情况和ECOG（Eastern Cooperative Oncology Group）评分；病理获取时间、分期、转移部位；化疗方案、最佳疗效、疾病进展时间、是否行胸部放疗；末次随访时间及死亡时间。采用电话形式进行随访，随访时间至患者死亡时间或2013年4月15日为止，存活时间以月为单位。

### 1.3 评定标准

根据实体瘤疗效评价标准（Response Evaluation Criteria in Solid Tumor, RECIST）评估疗效，分为完全缓解（complete response, CR）、部分缓解（partial response, PR）、稳定（stable disease, SD）和进展（progressive disease, PD）。有效率（RR）=（CR+PR）/全部病例数×100%。敏感复发是指初始治疗有效且初始治疗结束到疾病进展的时间 > 90天；耐药复发是指初始治疗结束到疾病进展的时间≤90天。总生存时间（overall survival, OS）指从病理获取时间至患者死亡或末次随访时间（月）。无进展生存时间（progression-free survival, PFS）指从病理获取时间至疾病进展或患者死亡的时间或末次随访时间（月）。

### 1.4 统计学处理

采用SPSS 18.0软件进行数据统计分析。采用*Kaplan-Meier*法计算患者的OS和PFS，并绘制生存曲线。采用单因素以及*Cox*回归多因素分析各种因素对生存期的影响。*P* < 0.05为差异有统计学意义。

## 2 结果

### 2.1 全组一般情况

查阅于2001年1月-2011年12月在北京协和医院住院治疗的SCLC患者的临床资料802例，根据实验对象入选标准，符合条件的广泛期SCLC患者共394例。全组患者年龄20岁-93岁，中位年龄为62岁。病理组织学上，经典SCLC 389例，混合癌5例。最常见的转移部位包括骨、胸腔积液、肝、脑和肾上腺。患者一般情况见表 1。

**1:** 全组广泛期小细胞肺癌患者的一般情况和总生存期的单因素分析 Clinical characteristics and univariate analysis of overall survival (OS) of all extensive-stage samll cell lung

Factor		*n* (%)	OS (month)	95%CI (month)	*χ*^2^	*P*
Sex					0.918	0.338
	Male	300 (76.1)	13.8	12.21-15.33		
	Female	94 (23.9)	16.5	14.36-18.64		
Age (yr)					6.936	0.008
	≥60	232 (58.9)	13.2	10.45-16.02		
	< 60	162 (41.1)	15.4	13.66-17.14		
Smoking history					2.269	0.132
	Yes	297 (75.4)	13.4	11.30-15.45		
	No	97 (24.6)	15.6	13.63-17.51		
ECOG PS					18.963	< 0.001
	0-1	304 (77.2)	15.6	14.00-17.14		
	2-4	90 (22.8)	9.9	5.90-13.96		
Brain metastasis					0.435	0.510
	Yes	71 (18.0)	11.8	5.92-17.68		
	No	323 (82.0)	14.9	13.30-16.50		
Liver metastasis					28.153	< 0.001
	Yes	78 (20.0)	9.3	7.60-11.00		
	No	316 (80.0)	16.4	15.16-17.64		
Bone metastasis					20.270	< 0.001
	Yes	128 (32.5)	10.0	8.26-11.80		
	No	266 (67.5)	16.6	15.32-17.82		
Adrenal metastasis					0.237	0.626
	Yes	62 (15.7)	14.5	11.82-17.20		
	No	332 (84.3)	14.8	13.12-16.54		
Malignant pleural fluid					0.045	0.832
	Yes	104 (26.4)	15.5	12.83-18.23		
	No	290 (73.6)	14.5	12.79-16.28		
Chemotherapy					41.372	< 0.001
	Yes	369 (93.7)	15.1	13.43-16.84		
	No	25 (6.3)	1.6	0.62-2.64		
Thoracic radiotherapy					14.017	< 0.001
	Yes	164 (41.6)	16.8	14.36-19.25		
	No	230 (58.4)	12.3	9.93-14.73		
ECOG PS: Eastern Cooperative Oncology Group performance status						

### 2.2 全组治疗情况

394例患者中25例未行化疗，369例接受化疗；164例患者进行了胸部放疗，91例患者在出现脑转移后进行了脑部放疗，8例行预防性全脑放射治疗。369例进行化疗患者的化疗方案包括EP或CE[顺铂（DDP）或卡铂（CBP）+依托泊苷（VP-16）]方案各75例和230例，其他化疗方案64例包括拓扑替康（TPT）+DDP方案12例、CODE[环磷酰胺（CTX）+阿霉素（ADM）/表阿霉素（EPI）+DDP+VP-16]方案10例、氨柔比星+DDP方案10例、紫杉醇（TAX）+DDP/CBP方案8例、替尼泊苷（VM26）+卡莫司汀（BCNU）方案4例、力比泰+DDP方案4例、口服VP-16方案4例、异环磷酰胺（IFO）+VP-16方案3例、吉西他滨（GEM）+DDP方案2例、伊立替康（CPT-11）+DDP方案2例、IFO+CBP方案1例、丝裂霉素（MMC）+DDP方案1例、VIP（VP-16+IFO+DDP/CBP）方案1例、CPE（CBP+TAX+VP-16）方案1例、COME[CTX+长春新碱（VCR）+甲氨蝶呤（MTX）+VP-16]方案1例。化疗1个-3个疗程者102例，4个-6个疗程者221例，7个疗程以上者46例；疗效PD者28例，SD者79例，PR/CR者197例，疗效不详者65例；化疗后耐药复发者158例，3个月-6个月复发者52例，6个月以上复发者31例，复发情况不详者128例；接受化疗的患者中126例接受了放射治疗。

### 2.3 全组生存情况及其影响因素

全组的中位总生存时间为14.8个月。1年、2年、5年生存率分别为58.9%、27.2%、7.8%。分析性别、年龄、吸烟情况、ECOG评分、转移部位、是否进行化疗、是否进行胸部放疗对全组394例广泛期SCLC OS的影响。单因素分析的结果表明广泛期SCLC的OS与年龄、ECOG评分、肝转移、骨转移、是否化疗、是否胸部放疗密切相关，其中未进行化疗患者的死亡风险是进行化疗的患者的4.919倍，若无肝、骨转移则死亡风险下降约50%；与其他因素如性别、是否吸烟、脑转移、肾上腺转移、有无胸腔积液的关系无统计学意义（表 1）。多因素分析的结果表明年龄、ECOG评分、肝转移、骨转移和是否化疗是广泛期SCLC OS的独立影响因素（表 2）。单因素分析结果显示广泛期小细胞肺癌OS与是否进行胸部放疗有关，但多因素分析结果显示OS与是否进行胸部放疗无关。

**2:** 全组广泛期小细胞肺癌OS的多因素分析 Multivariate analysis of OS in all extensive-stage SCLC patients

Factor		HR	95%CI	Wald	*P*
Age (yr)	< 60	0.68	0.52-0.90	7.565	0.006
ECOG PS	0-1	0.71	0.52-0.96	5.295	0.021
Liver metastasis	No	0.49	0.36-0.66	21.247	< 0.001
Bone metastasis	No	0.55	0.41-0.74	15.970	< 0.001
Chemotherapy	No	4.92	2.77-8.75	29.375	< 0.001
Compare with patients who were more than 60 years old, whose ECOG performance status 2-4, who were present with liver metastasis and bone metastasis and who received chemotherapy, respectively.					

### 2.4 一线化疗生存情况及其影响因素

接受一线化疗广泛期SCLC患者的中位OS为15.1个月。分析年龄、性别、ECOG评分、吸烟情况、不同转移部位、化疗方案、化疗疗效、化疗疗程数、是否进行胸部放疗、复发时间对一线化疗OS的影响。单因素分析的结果显示一线化疗广泛期小细胞肺癌的OS与年龄、ECOG评分、肝转移、骨转移、化疗疗效、化疗疗程数、胸部放疗、复发时间相关（表 3）。多因素分析的结果显示一线化疗广泛期SCLC的OS只与吸烟、骨转移、肝转移、化疗疗程数相关（表 4）。一线化疗的中位PFS为7.5个月。同样分析各因素对一线化疗PFS的影响。单因素和多因素分析的结果均显示一线化疗PFS与吸烟、肝转移、骨转移、化疗疗程数相关，单因素分析的结果显示一线化疗PFS与胸部放疗相关，但多因素分析的结果显示PFS与胸部放疗无关（表 3，表 5）。

**3:** 广泛期小细胞肺癌一线化疗OS和PFS的单因素分析 Univariate analysis of OS and PFS in extensive-stage SCLC patients who received first-line chemotherapy

Factor		*n*	OS (95%CI)	*χ*^2^	*P*	PFS (95%CI)	*χ*^2^	*P*
Sex				2.520	0.112		4.058	0.044
	Male	285	14.3 (12.66-15.94)			7.2 (6.70-7.70)		
	Female	84	17.0 (13.19-20.75)			7.8 (5.27-10.33)		
Age (yr)				5.406	0.020		0.268	0.605
	≥60	213	13.4 (10.81-15.93)			7.3 (6.78-7.82)		
	＜60	156	15.6 (13.71-17.43)			7.7 (7.08-8.38)		
Smoking history				3.736	0.053		7.335	0.007
	Yes	280	14.1 (12.08-16.13)			7.2 (6.62-7.78)		
	No	89	16.8 (14.36-19.24)			8.4 (5.87-10.99)		
ECOG PS				7.664	0.006		2.112	0.146
	0-1	297	15.6 (14.05-17.10)			7.6 (6.89-8.31)		
	2-4	72	13.0 (9.01-16.99)			7.2 (6.42-7.92)		
Brain metastasis				1.369	0.242		0.013	0.909
	Yes	67	11.8 (5.92-17.68)			7.6 (6.92-8.22)		
	No	302	15.4 (13.71-17.09)			7.4 (6.89-7.80)		
Liver metastasis				22.794	< 0.001		7.879	0.005
	Yes	71	9.4 (7.61-11.19)			6.9 (5.10-8.64)		
	No	298	16.5 (15.23-17.77)			7.7 (7.14-8.32)		
Bone metastasis				23.212	< 0.001		20.231	< 0.001
	Yes	118	10.2 (8.33-12.01)			5.4 (4.21-6.53)		
	No	251	16.8 (15.03-18.57)			8.1 (7.33-8.81)		
Adrenal metastasis				0.109	0.741		0.694	0.405
	Yes	61	14.9 (11.35-18.45)			7.9 (6.96-8.90)		
	No	308	15.4 (13.44-17.30)			7.5 (6.98-7.96)		
Malignant pleural fluid				0.827	0.363		0.800	0.371
	Yes	95	15.6 (13.01-18.13)			7.6 (6.81-8.39)		
	No	274	14.8 (12.99-16.67)			7.5 (6.93-8.01)		
Chemotherapy regimen				4.956	0.084		0.716	0.699
	CE regimen	230	14.9 (13.12-16.68)			7.7 (6.95-8.45)		
	EP regimen	75	17.4 (13.23-21.51)			7.2 (6.14-8.26)		
	Other regimens	64	11.5 (9.20-13.80)			7.4 (6.40-8.34)		
Curative effect				62.003	< 0.001		145.099	< 0.001
	PD	28	5.9 (3.48-8.32)			2.0 (1.39-2.61)		
	SD	79	13.4 (9.82-16.92)			5.9 (4.28-7.52)		
	PR/CR	197	16.6 (15.21-17.93)			8.2 (7.51-8.96)		
	Unknown	65	10.0 (7.20-12.80)			8.4 (5.23-11.63)		
Cycle number				67.903	< 0.001		84.932	< 0.001
	1-3	102	7.7 (5.62-9.78)			3.2 (2.53-3.93)		
	4-6	221	16.4 (14.93-17.87)			7.6 (7.08-8.06)		
	≥7	46	16.6 (14.50-18.64)			10.6 (8.90-12.36)		
Thoracic radiotherapy				14.557	< 0.001		10.744	0.001
	Yes	164	18.6 (14.51-22.75)			8.9 (7.80-9.95)		
	No		13.1 (10.94-15.20)			6.9 (6.00-7.74)		
Recurrence status				37.415	< 0.001		170.031	< 0.001
	Resistant	158	11.6 (9.96-13.31)			5.1 (4.71-5.54)		
	3-6 months	52	18.5 (14.38-22.68)			12.8 (11.52-14.14)		
	≥6 months	31	24.9 (11.30-38.56)			13.2 (8.96-17.51)		
	Unknown	128	13.2 (8.96-17.51)			8.4 (7.61-9.25)		
EP: etoposide+cisplatin; CE: carboplatin+etoposide; PD: progressive disease; SD: stable disease; PR: partial response; CR: complete response.									

**4:** 广泛期小细胞肺癌一线化疗OS的多因素分析 Multivariate analysis of OS in extensive-stage SCLC patients who received first-line chemotherapy

Factor		HR	95%CI	Wald	*P*
Smoking	Yes	1.39	1.01-1.91	4.156	0.041
Liver metastasis	Yes	2.20	1.57-3.07	20.952	< 0.001
Bone metastasis	Yes	1.70	1.26-2.28	12.27	< 0.001
Cycle number				43.771	< 0.001
	≥7	0.22	0.14-0.36	37.592	< 0.001
	4-6	0.39	0.28-0.55	29.696	< 0.001
Compare with patients who did not smoke, who were absence of bone metastasis and liver metastasis, whose cycle number was 1-3, respectively.					

**5:** 广泛期小细胞肺癌一线化疗PFS的多因素分析 Multivariate analysis of PFS in extensive-stage SCLC patients who received first-line chemotherapy

Factor		HR	95%CI	Wald	*P*
Smoking history	Yes	1.52	1.16-2.01	8.878	0.003
Liver metastasis	Yes	1.63	1.22-2.20	10.549	0.001
Bone metastasis	Yes	1.64	1.28-2.11	14.988	< 0.001
Cycle number				47.159	< 0.001
	≥7	0.25	0.17-0.38	44.261	< 0.001
	4-6	0.48	0.36-0.64	10.262	0.001
Compare with patients who did not smoke, who were absence of liver metastasis and bone metastasis, whose cycle number was 1-3, respectively.					

### 2.5 不同年份一线化疗OS的比较

将369例进行化疗的患者按诊断时间的不同分为3组，即2001年-2004年组80例、2005年-2008年组152例、2009年-2011年组137例，3组的OS和95%可信区间分别为11.6（6.55-16.72）、15.0（12.56-17.44）和15.6（13.04-18.10）个月，无明显统计学差异（χ^2^=4.338, *P*=0.144）。3组的生存曲线见图 1。

**1:**
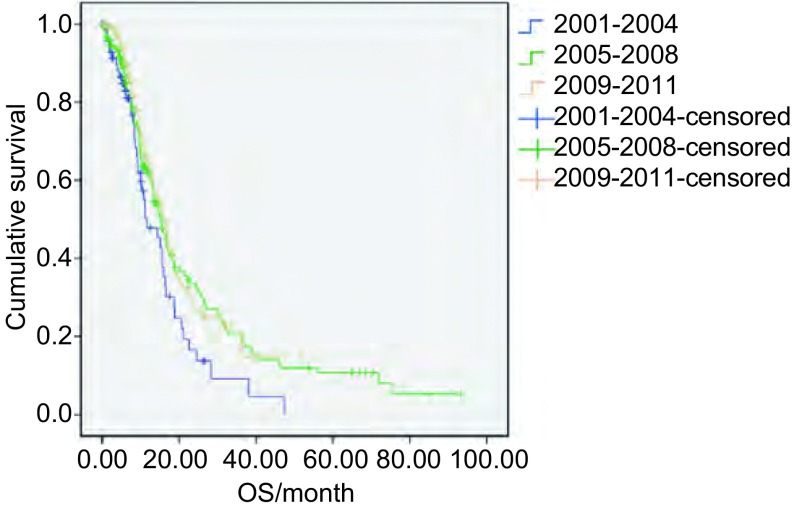
不同年份广泛期小细胞肺癌一线化疗OS的比较 Comparison of OS in extensive-stage SCLC patients who received first-line chemotherapy of different years. OS: overall survival; SCLC: small cell lung cancer.

## 3 讨论

本研究全组中位OS为14.8个月，1年、2年、5年生存率分别为58.9%、27.2%、7.8%，一线化疗中位OS为15.1个月，中位PFS为7.5个月，与文献报道基本一致^[2, 3]^，另外，3个时间段OS的比较提示广泛期SCLC的生存情况未得到明显改善。全组OS与年龄、ECOG评分、肝转移、骨转移、是否化疗密切相关。一般SCLC多于中老年发病，该研究394例患者的年龄跨度为20岁-93岁，中位年龄为62岁。年龄 < 60岁死亡风险较年长者下降32%，这可能与年龄较轻的患者一般状况较好、合并的全身性疾病少，更能耐受化疗有关。多项研究显示ECOG评分明显与SCLC的预后相关^[5]^，该研究同样证实了该观点。广泛期SCLC的治疗核心是化疗，未接受化疗的广泛期SCLC的生存期只有1个月-3个月，化疗比最佳支持治疗（best supportive care, BSC）能延长生存期^[6]^。本研究中进行化疗的患者的中位总生存时间为15.1个月，未进行化疗的中位总生存时间为1.6个月，未进行化疗患者的死亡风险是进行化疗的患者的4.919倍，故广泛期SCLC患者应积极接受化疗。另一个影响预后的因素是转移部位，有研究称肝转移与预后明显相关^[3]^，而且转移部位越多预后越差^[7]^，本研究证实若无肝、骨转移则死亡风险下降约50%。

若SCLC患者接受一线化疗，则OS、PFS都与化疗疗效、化疗疗程数相关，与化疗方案无关。首先，化疗疗效是预测广泛期SCLC预后的重要指标。本研究达到PR/CR患者的中位OS和PFS分别为16.6个月和8.2个月，PD的中位OS和PFS分别为5.9个月和2.0个月，即PR/CR患者较PD患者的OS延长1年、PFS延长半年，死亡风险下降62%，疾病进展风险下降85%，故如果化疗疗效能达到PR将有益于生存。这与临床经验相一致，因为如果疗效达到PR说明患者对化疗敏感，而如果是PD则提示耐药。而达到SD的患者的OS为13.4个月，与PR/CR患者的OS比较无统计学意义（χ^2^=5.512, *P*=0.019 > *α*’=0.05/6=0.008），即达到SD患者的生存时间并没有明显差于PR/CR的患者。其次，化疗疗程数同样可以影响OS和PFS。单因素结果显示化疗1个-3个疗程者的OS和PFS只有7.7个月和3.2个月，因为一般化疗1个-3个疗程者一般状况较差或者对化疗药物不敏感；化疗4-6疗程和≥7疗程的中位OS差别不大（χ^2^=2.706, *P*=0.100），而两者的中位PFS有明显差异（χ^2^=14.580, *P* < 0.001）；Bozcuk^[8]^的*meta*分析也提示增加化疗周期（即维持治疗）只能增加中位进展时间，却没能明显改善生存，故适合的化疗疗程数是4个-6个疗程。值得注意的是胸部放疗在全组OS、一线化疗的OS和PFS的单因素和多因素分析结果是不一致的，有关胸部放疗在广泛期SCLC治疗中的作用有待进一步研究。

综上所述，年龄 < 60岁、体能状况好、无肝、骨转移的广泛期SCLC患者预后更好。广泛期SCLC患者应积极进行化疗，一线化疗的化疗疗效达到SD、PR-CR有益于生存；适合的化疗疗程数目是4个-6个疗程。
